# Editorial: Cell organelle exploitation by viruses during infection, volume II

**DOI:** 10.3389/fmicb.2023.1251168

**Published:** 2023-09-12

**Authors:** Parikshit Bagchi

**Affiliations:** Department of Cell and Developmental Biology, University of Michigan Medical School, Ann Arbor, MI, United States

**Keywords:** virus, cell, host-virus interaction, cellular organelle, virus replication cycle

Viruses exploit host machinery to replicate inside host cells. The present Research Topic is focused on how different cellular organelles and proteins are utilized by viruses for successful infection.

In this Research Topic, the first article by Wang et al. showed how a host protein N4BP3 (NEDD4 Binding Protein 3) regulates the innate immune response elicited by RNA viruses. The RIG I-mediated innate immune response plays a very important part in antiviral responses against RNA viruses. In this article, the authors demonstrated that N4BP3 is a critical component of the RIG-I-like receptor (RLR)-mediated innate immune response by targeting the mitochondrial antiviral signaling (MAVS) protein.

Calcium is an important trace element in animals and is an initiator and regulator of a variety of intra- and extracellular physiological pathways, which are also targeted by viruses. In the review article by Qu et al., the authors discussed how a wide variety of viruses exploit calcium channels during almost all phases of their virus life cycle from adsorption, penetration, and uncoating to virus replication and release.

In the next article included in the Research Topic, Chen et al. showed how infectious spleen and kidney necrosis virus (ISKNV) regulates the host apoptosis pathways by manipulating reactive oxidative species/Nrf2-mediated oxidative stress response for the regulation of mitochondrion-mediated Bax/Bak cell death signals in GF-1 cells. The authors hypothesized that ISKNV triggered reactive oxidative species (ROS)/Nrf2 stress signals, which either modulate the apoptotic Bax/Bak death pathway or reduce viral replication in fish cells.

Finally, the review article by Fu et al. explained how mitophagy plays an important role in virus infection. Mitophagy is an important cellular process in which phagosomes select damaged and excess mitochondria, which are subsequently degraded by autophagic autophagolysosomes. Mitophagy controls the quality of mitochondria and maintains normal cellular function and physiological processes. Different viruses utilize various strategies to activate or inhibit mitophagy for their replication or regulation of host innate immune response and apoptosis. HCV and HBV regulate mitophagy through the PINK1/Parkin-dependent pathway. Viruses such as the measles virus (MEV), HHV-8, CSFV, EBV, HBV, HCV, IAV, SARS-CoV-2, and HPIV3, antagonize IFN-I production through mitophagy and promote self-replication. Another way to promote viral self-replication is through triggering mitophagy to suppress inflammation and inhibit apoptosis. This study has shown that some viruses such as MeV and IAV inhibit the activation of the NLRP3 (NLR family pyrin domain containing 3) inflammasome by inducing mitophagy, thereby helping the virus to escape host immune defenses. Whereas viruses such as HBV, HCV, VEEV, CSFV, PRRSV, NDV, and TGEV can all induce mitophagy through different mechanisms and inhibit apoptosis for persistent infection.

The contents of this Research Topic emphasized and elaborated on how important cellular pathways are hijacked by different viruses and utilized in every aspect of their infection cycle. These host factors can be used as targets for drug development and, as it seems that different viruses are utilizing common host factors, it can be possible to use one drug for multiple viruses.

## Author contributions

PB wrote the editorial.

